# Moving from ideas to action - developing health financing systems towards universal coverage in Africa

**DOI:** 10.1186/1472-698X-12-30

**Published:** 2012-11-08

**Authors:** Laurent Musango, Juliet Nabyonga Orem, Riku Elovainio, Joses Kirigia

**Affiliations:** 1 Health Systems and services cluster, WHO Africa Regional office, 06 Citée de Djoué, Brazzaville, Congo; 2 Health Systems and services cluster, WHO Uganda office, Kampala, Uganda; 3 Department of Health Systems Financing, WHO, avenue Appia 20, 1211, Geneva 27, Switzerland

**Keywords:** Health financing, Ministry of health, Ministry of finance, Africa, Panel discussion

## Abstract

**Background:**

Accelerating progress towards universal coverage in African countries calls for concrete actions that reinforce social health protection through establishment of sustainable health financing mechanisms. In order to explore possible pathways for moving past the existing obstacles, panel discussions were organized on health financing bringing together Ministers of health and Ministers of finance with the objective of creating a discussion space where the different perspectives on key issues and needed actions could meet. This article presents a synthesis of panel discussions focusing on the identified challenges and the possible solutions. The overview of this paper is based on the objectives and proceedings of the panel discussions and relies on the observation and study of the interaction between the panelists and on the discourse used.

**Summary:**

The discussion highlighted that a large proportion of the African population has no access to needed health services with significant reliance on direct out of pocket payments. There are multiple obstacles in making prepayment and pooling mechanisms operational. The relatively strong political commitment to health has not always translated into more public spending for health. Donor investment in health in low income countries still falls below commitments. There is need to explore innovative domestic revenue collection mechanisms. Although inadequate funding for health is a fundamental problem, inefficient use of resources is of great concern. There is need to generate robust evidence focusing on issues of importance to ministry of finance. The current unsatisfactory state of health financing was mainly attributed to lack of clear vision; evidence based plans and costed strategies.

**Discussion:**

Based on the analysis of discussion made, there are points of convergence and divergence in the discourse and positions of the two ministries. The current blockage points holding back budget allocations for health can be solved with a more evidence based approach and dialogue based on a clear vision and costed strategic plan articulated by the ministry of health. Improving health in Africa is a driver for long-term economic growth and development and this is the reason why the ministries of health and finance will need to find common ground on how to create policy coherence and how to articulate their respective objectives.

## Background of panel discussions between MoH and MoF

Several African countries have recently implemented health financing reforms and actions that have improved coverage of health services, especially for the most vulnerable individuals [1-3]. However, these positive developments in some countries should not mask the reality that by and large, universal coverage - defined as ensuring access to needed, effective health services for the whole population without putting individuals at risk of financial hardship as a result of paying for those services is still a far-away objective for most countries of the African Region [1]. Failing to move towards universal coverage will also hamper countries’ possibilities to reach the health MDGs, other crucial health objectives and targets beyond year 2015 [2,4-6]. Accelerating progress towards universal coverage calls for concrete actions that reinforce social health protection through establishment of sustainable health financing mechanisms. There are many obstacles faced in the implementation of needed reforms and actions; some are financial, some administrative, some political, and most often countries face a mix of problems and challenges.

In order to explore possible pathways for moving African countries past the existing obstacles and bottlenecks, and towards universal coverage, three panel discussions were organized on health financing. These panels gathered together Ministers of health and Ministers of finance with the objective of creating a discussion space where the different perspectives on the key issues and needed actions could meet.

The first panel discussion took place on 24 July 2010 in Kampala as part of the 15^th^ Ordinary session of the Assembly of the African Union (AU). The panel included the Minister of Health of Ethiopia, the Minister of Finance of Sierra Leone, the Regional Director of the WHO Regional Office for Africa, the acting Human Development Director of the World Bank, and the Director of Partnerships at the Global Fund to Fight AIDS, Tuberculosis and Malaria (GFATM). The stated objectives of the panel, organized as a Side Event of the AU assembly, were to (i) identify the reasons for the current poor state of health financing in Africa and define what could be done by both African and global stakeholders to improve the situation; and (ii) make a case for health investment and financing, especially within a continuum of care for maternal, infant and child health.

The second panel discussion took place on the 28^th^ March 2011 in Addis Ababa as part of the 4^th^ Joint Annual Ministerial Meeting of the AU Conference of Ministers of Economy and Finance and the East Central Africa Conference of African Ministers of Finance, Planning and Economic Development, the panel included the Minister of Health from Senegal and two Ministers of Finance (Sierra Leone and Cameroon); WHO Regional Director for Africa and AU Commissioner for Social Affairs participate as moderators. Twelve delegates of Ministers of Health and all Ministers of Finance or their delegates from all AU countries participated in this panel and contributed during the back to back question and discussion session. The objectives of this panel discussion were: (i) to share country experiences in securing sufficient funding for strengthening health systems and increasing access to quality health care towards achieving the MDGs; (ii) to share and disseminate the Harmonization for Health in Africa (HHA) report on “Investing in Health for Africa: the case for strengthening systems for better health outcomes”; (iii) to discuss the main funding strategies and options for increasing fiscal space to strengthen national health systems in the African Region; and (iv) to propose ways of raising additional funds for the health sector and improving the efficiency of utilization of both domestic and external resources.

The third panel discussion took place on 30^th^ September 2011 during the 61^st^ session of the World Health Organization (WHO) Regional Committee in Côte d’Ivoire, Yamoussoukro [1]. The panel discussion comprised of four ministers of finance and three ministers of health. The panel discussion was chaired by the Minister of Health of Senegal and co-chaired by Regional Director of UNICEF/West and Central Africa and UNFPA Regional Director. The WHO Director General and Regional Director for Africa took part in the panel discussion. Several Ministers of Finance and Ministers of Health or their delegates from all the 46 countries of the WHO African Region participated in the panel session and contributed to the discussions. The objectives of this panel discussion were similar to the second panel discussion: (i) to share country experiences; (ii) to disseminate the HHA report on Investing in Health for Africa; (iii) to discuss key funding strategies and options for increasing the tax base; and (iv) to propose ways for raising additional funds for the health sector and improving efficiency in the utilization of both domestic and external resources.

Several themes and questions were highlighted during the panel discussions, this paper will focus on four key issues: (i) lowering financial barriers to access to health care by improving and extending prepaid and pooling mechanisms including the question of user fee exemptions; (ii) mobilization of domestic and international resources for health; (iii) efficiency and equity in the use of resources including incentive for health workers; and (iv) making evidence based health financing policy decisions. These specific issues are highlighted here since they offer some of the most important entry points in the dialogues between ministries of health (MoH) and ministries of finance (MoF) and as such they were also central in all the different panel discussions. They represent issues around which the policy articulation between these two ministries is particularly important; for example, as the discussions revealed, the question of extending coverage from prepaid and pooled sources was often looked at from the angle of public financial management by the MoF, which is not at all a natural “instinct” for the MoH and thus stirred some interesting discussion on how to go about in assuring that the increased funding that should be channeled in the pooling mechanisms will be used in an efficient and accountable manner.

The purpose of this article is to present and synthesize the panel discussions focusing on the identified key challenges and on the possible solutions to address them through strengthening the collaboration between the MoH and MoF in the area of health financing. Although the discussion in this document mainly revolves around the interaction and policy articulation between the MoH and the MoF, it is evident that, depending on the context, several other ministries such as the Ministry of planning or the Ministry of social affairs will be central actors when it comes to health financing policy. In order to push forward, plan and successfully implement the key health financing reforms and actions, far reaching support and participation from all the stakeholders including Heads of State, parliamentarians, non-government entities and a host of other actors is needed.

As the issues raised during the panels reveal, the focus of the discussions was on health financing areas where the overlap between ministries of finance and ministries of health is evident. For example, when it comes to raising more (sustainable) domestic funds for health, it is clear that without a strong coordination and enhanced collaboration between the MoH and the MoF this objective will not be reached. Ultimately, most, if not all, health financing actions and reforms that effectively have the potential to move countries towards universal coverage will need strong collaboration and policy articulation between these two ministries. The different initiatives aiming at providing free care at the point of service delivery serve as concrete and illustrative examples of the importance of this coordination. In the past, the user fee abolition or exemption policies have not been successful without a clear convergence of actions and strategies from the ministries of health and finance [7,8] - mainly in order to secure a functional financial compensation mechanism that can replace the loss of revenue from user fees to health facilities and can counter the distortions provoked on staff incentives. In some countries where the user fee exemption or abolition policies were not jointly planned and implemented there have been important weaknesses in the reimbursements to the facilities; this has lead to stock outs of medicine and decrease of staff morale [9,10].

This paper relies on the observation and study of the interaction between the panelists and on the discourse used. The approach of this paper borrows from the discourse analysis methodology, which studies the reasoning behind views expressed in a particular context by different actors. Particularly interesting to the analysis are the points of convergence and points of divergence that can be detected in the discourses of the different participants and commentators. This will then guide this paper towards an analysis of the type of convergence existing, but, maybe more interestingly, also on the implicit and explicit reasons of the discordances. Ultimately this analysis aims at producing an insight into how to build on the existing convergence and to smoothen out the divergence between the MoH and the MoF in order to design solutions to some of the key health financing problems.

## Key Heath financing issues discussed

### Financial barriers to accessing heath care

**Figure 1:**
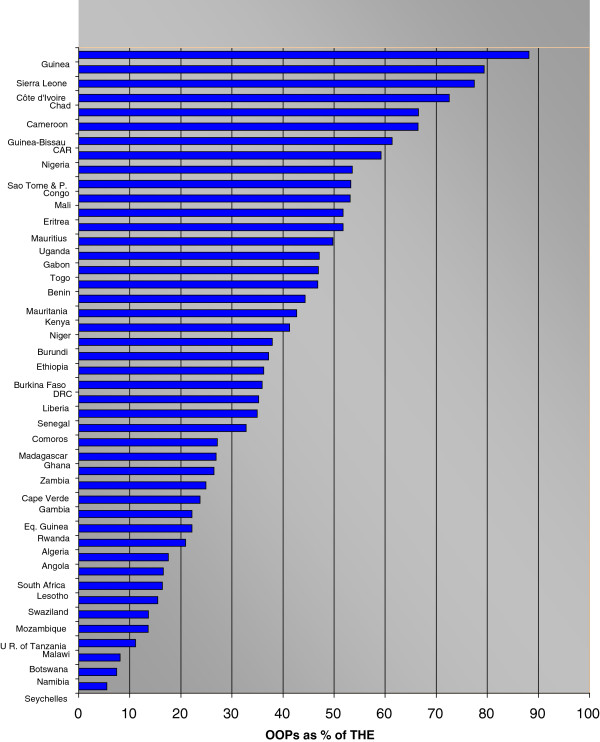
Out-of-pocket (OOP) expenditure as % share of total health expenditure (THE) WHO AFRO countries 2009

Currently a large proportion of the African population has no access to needed health services because they cannot afford to pay for them or because these services are not available in the first place [4,11]. Moreover many of those who do access the health services they need and have to pay for them out of their pocket are pushed into poverty [10,12]. The latest National Health Accounts (NHA) data show that in 13 of the 46 countries in the WHO African region out-of-pocket (OOP) spending exceeds 50% of total health expenditure (THE), while the (un-weighted) average in the region stands at 38.3% - see Figure 1. In 38 out of 46 countries, the level of private prepaid plans expressed as a percentage of private expenditure is less than 10% [13]. Where OOP is the norm, poor households tend to borrow money at exorbitant interest rates, sell assets, and may take children out of school after settlement of bills for one episode of illness [14,15]. These catastrophic health expenditures^a^ often push whole families below the poverty line. This situation of impoverishment and financial hardship from health payments and the subsequent illness-poverty cycle is an important obstacle for economic development. It is expected that universal access to health care should lead to overall human development, and therefore, economic development. Increasing total health expenditure using OOP cannot ensure universal coverage. It is well understood that in order for countries to reach universal coverage, financial barriers to access to health services need to be lowered, more funds need to be raised and managed through prepayment and pooling mechanism, and a particular focus should be put on enhancing coverage of the poorer and vulnerable population groups.

During the panel discussions the participants acknowledged the need to move from direct OOP health spending towards prepaid and pooled mechanisms. This strategic direction should come under the objective of covering those population groups who cannot directly contribute by ensuring cross-subsidization in health financing between population groups including the community level. It was acknowledged that this would not only cushion households against catastrophic out-of-pocket expenditures on health but also lead to absolute increases in funds available for health and to more efficiency in the collection of resources. However, it was noted during the discussions that there has been, and often still are, multiple obstacles in making the prepayment and pooling mechanisms operational. Controlling the fiduciary risk (including mismanagement, waste and fraud) for example is one of the major challenges in implementing and managing different types of health financing schemes and mechanisms relying on prepayment and pooling. While this public finance management issue is relevant to all levels of health financing systems, it was argued that a particular focus should be put on schemes that are administered at the community level or are in one way or another outside of the general public administration system. The major problem, especially from the MoF point of view, is that their management and financial control relies on people who largely do not have the financial management capacities required.

This reveals that in order to keep the MoF engaged in different health financing reforms and actions that aim at reinforcing and building prepayment and pooling mechanisms, a focus on financial management and on transparency and accountability of these mechanisms and schemes– at all levels - are clear prerequisites. The panel discussions concluded that financial management capacities, including competencies in budgeting, planning, accounting, auditing, and monitoring and evaluation need to be reinforced at all operational levels of the health financing system. This should apply to institutional entities such as social insurance funds; community based insurance schemes or local level public administration entities handling health funds allocated from the national budget. This underscores the need for an enhanced dialogue between the MoH and MoF in order to overcome the current managerial obstacles, build relevant capacities and to build trust that the health financing mechanisms that effectively replace direct payments by prepaid and pooled funds use public resources in an effective and transparent manner.

It was also noted that while these efforts to strengthen financial management in the health sector should be undertaken within a government- and administration-wide approach there is also a case for the health sector to be an actor in wider public finance management reforms. Resources for health are mobilized within government wide expenditure frameworks. Thus the benefits of a dialogue between the MoH and the MoF on public finance management could be wider than just the impact on the health sector.

Many African countries have implemented different types of user fee exemptions and/or abolition of fees for health during the last decade and increasingly in the last few years [9,11,16,17]. These have been important initiatives for lowering financial barriers to care and have been often followed by spectacular increases in health service utilization. However, it was noted during the panel discussions that, these will fall short of their objective if not integrated in a larger strategy of increasing prepayment and pooling. The participants noted the suboptimal performance of exemption mechanisms in majority of cases emphasizing that user fee abolition and exemption arrangements need to be interim measures as we move towards prepayment pooled mechanisms. Concretely this means that if user fees are abolished (for all or for a selected population group), a mechanism needs to be put in place in order to fill the gap in revenues lost. It is only by implementing some type of compensation mechanism and assuring its sustainable funding that the systemic objective of increasing the share of prepaid and pooled funds can be reached. Here again, the linkage between the MoH and the MoF is crucial since the user fee exemption and abolition policies will not be effective without a joint strategy that is supported by both ministries. The MoH should also keep in mind that in order to gain MoF’s engagement in a user fee exemption/abolishment policy, it needs to provide realistic and evidence based projections of how much money is needed for compensating the revenue losses. Both ministries need to also be involved in planning and implementing the strategies and mechanism that will be employed to allocate and channel funds, as sometimes it is not only a question of how much is being allocated as compensation but also how this compensation is channeled and used.

Finally, it was also concluded that the current situation regarding coverage from prepaid and pooled funds is too much tilted towards the well-off population groups mainly working in the formal sector which often represents only a small proportion of the economy of a typical African country. Those belonging to the informal sector and/or whose activities mainly consist of small scale agriculture are often left out - because they not covered by any specific contribution based scheme since they are not able to contribute. In addition, public (thus prepaid and pooled) funding available that is channeled directly to health services delivery is often skewed to high level care in urban areas. Aligning public health funding priorities with the primary health care (PHC) approach, channeling public funds through insurance mechanisms for covering those who cannot directly contribute and using a community based approaches for identifying and targeting those who would need support the most were seen as key strategies for extending coverage to the most vulnerable sector of the society.

### Mobilization of domestic and international resources

Per capita health expenditure in Africa is the lowest worldwide and expenditure for maternal and child health are particularly inadequate. This fact was strongly echoed during the panel discussions. This alarming observation catalyzed discussions that were revolving around different options of raising more resources for health. Average total per capita expenditure on health for low income countries in the WHO AFR stands at US$26 compared to US$183 for EMRO and US$2,204 for EURO [18].

One of the key questions discussed was how to translate economic growth into resources for health and particularly for health systems strengthening. It was noted that in Africa the relatively strong political commitment to health sector development has not always translated into more public spending for health. Reaching the health expenditure objectives has been often difficult because of inadequate allocation and further compounded by budgetary cuts that have not spared the health sector. As this has happened during a period of strong economic growth, in average, on the continent there were some voices that questioned the logic of tightened budgetary control on public health spending which eventually has resulted in overall low coverage rates – especially for the most vulnerable population groups who will not be able to access services without public health funding. The discussions did not reach any clear consensus on why economic growth has not translated, in average, into more public health spending (at least in relative terms), but one of the general assumptions is that countries will not be able to substantially increase public health spending without health financing system reform that can break and/or circumvent some of the structural rigidities (related to for example rules on budget allocation). Countries such as Ghana or Rwanda have shown that with a comprehensive health financing strategy that translates into reform and actions, countries can create structures and channels that enable substantial increases in investment in health.

The 2001 Abuja declaration was used as an illustration of the recent problems in generating domestic resources for health. The Abuja declaration set a target for African Union countries of allocating “at least 15%” of national budgets to the health sector; this target was achieved by only 5 countries in 2010 as shown in Table 1. Meanwhile, thirteen countries had reduced the relative contribution of government allocation to health during the same period. In the four countries where there was no notable upward or downward trend, the average amount allocated to the health sector was 9. 8% [18]. However, it is important to note that allocations to the health sector ranged from as low as 2% to as high as 20% [18]. There are surely many reasons behind these notable differences, but as already discussed there are most probably structural reasons, and for many countries the relatively low priority given to health spending from public funds could relate to a “stagnation” of the health financing mechanism - and the health system in general - compared to other, maybe more dynamic sectors that are able to attract and absorb more funding than the health sector.

**Table 1:** Countries’ performance against the Abuja target

	**Abuja commitment GGHE%GGE > 15%**	**Abuja commitment GGHE%GGE < 15%**
THE per capita > 44US$	Botswana, Rwanda, Zambia	Algeria, Angola, Cameroon, Cape Verde, Congo, Côte d'Ivoire, Equatorial Guinea, Gabon, Ghana, Guinea-Bissau, Lesotho, Mauritius, Namibia, Nigeria, Sao Tome and Principe, Senegal, Seychelles, Sierra Leone, South Africa, Swaziland, Uganda
THE per capita < 44US$	Madagascar, Togo	Benin, Burkina Faso, Burundi, Central African Republic, Chad, Comoros, Democratic Republic of the Congo, Eritrea, Ethiopia, Gambia, Guinea, Kenya, Liberia, Malawi, Mali, Mauritania, Mozambique, Niger, United Republic of Tanzania
TOTAL	5	40

*GGEH*: Government general expenditure on health; *TGE*: Total government expenditure; *THE*: Total health expenditure.

Member states emphasize that, it is logical to consider both the Abuja Declaration target of 15% of the government budget allocated to the health sector and the recommendation of the High-level Taskforce on Innovative International Financing for Health Systems to allocate at least USD44 per capita to deliver an essential package of health services [19]. Over a third of African Union countries have not managed to raise health spending to the level of US$44. Only three countries have managed to meet both targets that is, allocating 15% of the national budget and also managed to reach the USD44 per capita [18].

The current funding shortfalls in many African countries emphasize the need for donor countries and other development partners to continue investing in health in low income countries. It was also noted that it would be possible to achieve a large increase in available international resources for health if the donor countries would fulfill their promise to allocate 0.7% of their GNI in official development assistance (ODA). There were also discussions around the concern of donor funding being unpredictable and not harmonized with national priorities and mechanisms. A sector wide approach (SWAp) was mentioned as a solution for better coordination and harmonization between development partners themselves and between development partners and the countries. The SWAp mechanism was seen as a main entry point that would allow channeling of external funds through activities included in the national health plans (NHP). There was a clear convergence between the MoH and MoF around the issue of aid harmonization and alignment. It was felt that keeping external funding “on budget” allows MoH more control over allocating these funds towards the priority areas as identified in sector National health plans. There could thus be a case for a joint MoH/MoF advocacy towards donors to enforce concrete actions that follow the spirit and content of the Paris Declaration on Aid Effectiveness and the Accra Agenda for Action.

Panel participants emphasized that external funds should only play a catalytic role with the bulk of funding for health coming from domestic sources. This raises the need for innovative domestic revenue collection mechanisms in order to effectively increase expenditure on health. The question of revenue collection for health is an area which naturally lies within the remit of the MoF with the MoH only playing a marginal role. The panel discussions showed that there were several convergence points between the MoF and MoH thinking. For example, there was a consensus around the need to explore some innovative options such as using remittances and other foreign direct investment (FDI) based financing mechanisms. Another source of possible additional funds that was discussed was the possible utilization of taxes and levies on products such as tobacco and alcohol. The health advocates often push for these "sin taxes" since they have a direct public health effect through the reduction of consumptions of these products; but as they are also sources of additional public revenues the MoF is also often keen on implementing them. This is a convergence in the objectives of the two ministries that could be used more powerfully in many contexts to push for levies and taxes that can have a positive public health aspect but can also be a way to raise more funds for health. The paradox between the objectives of raising more revenue for health and lowering the consumption of the products must of course be balanced out; but it is still possible to reach both of these objectives if one takes into account the current estimations of price elasticity. On the other hand if the consumption would dramatically drop and the revenues fall, this would still be beneficial outcome from the financial perspective since the cost savings from decreasing cases of cancers, cardiovascular diseases and other conditions would be tremendous. This is also an argument that the MoH should provide to MoF when discussing “sin taxes”. However, there is often a divergent view between MoH and MoF on earmarking of these funds and MoH does not always get through its view that money raised through these mechanisms should be earmarked to health. Eventually MoH along with other health actors will need to make a solid case for health investment and financing. As we shall see in the following sections, making health spending more efficient and relying on health financing strategies and actions that are based on a clear vision and on evidence are some of the most important aspects in this dialogue between MoH and MoF. It has to be underlined also that many of the decisions of resource allocation are political; hence making the case for health investment should be also extended to the political actors such as parliamentarians and Heads of State.

### Efficiency and equity in use of resources in the health sector

The panel discussion brought up the notion that while inadequate funds for health were a fundamental problem in the African region, inefficient use of resources was also a great source of worry. There was a large consensus that the achievement of national and international health development targets requires not only increased funding, but also efficient use of existing resources and greater equity in financing and accessing quality-health care.

Inefficiency in management of health system and its subsystems is well known. At country level, weak policy setting environment leads to weak national health planning that in turn leads to a multiplication of actions that may not be comprehensive and harmonized. The legal and regulatory frameworks are inadequately reinforced and as result inappropriate procurement, loss and irrational use of medicines; inappropriate staff mix and deployment coupled with a lack of performance incentives are not uncommon. There are also weak policies related to allocation and timely disbursement of funds to the end users. This may lead to overuse and overfunding of certain health services and avoidable wastages especially due to pilferage. WHO estimates that globally, 20-40% of all health spending is wasted through inefficiency [11]. This is a global estimate and countries need to have country level estimations of the level of inefficiency. The challenge that remains is coming up with comprehensive and robust methodologies to measure efficiency at a sector level. However, governance in some countries is commendable in that they are able to achieve more and better results than others at the same or higher level of health expenditure - demonstrating the huge potential for efficiency gains.

**Figure 2:**
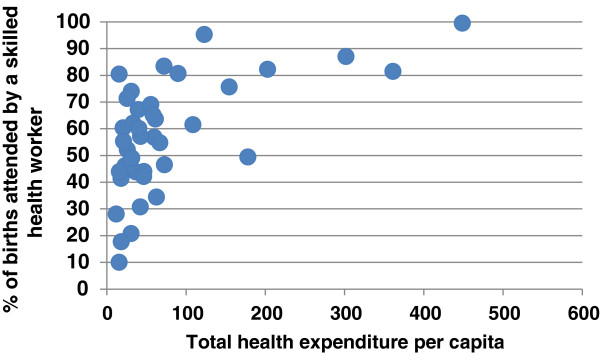
**Countries’ attainment of outputs for a given level of expenditure.** WHO AFRO countries 2009

In Figure 2, we see that some countries achieve more than others with the same level of health spending, which indicates that there is ample scope for efficiency gains.

One important issue that was raised in the panel discussions was the relatively high spending on medicines and other medical goods. It was noted that at the national level one of the problems was the low capacity to produce essential medicines and commodities such as insecticide-treated bed nets. Some of the obstacles related to this problem come from international trade agreements and some other relate to the low levels of economic development in general. Solving these problems will need general economic development and trade policy approach but the MoH should still take part in these policy dialogues. Another solution for increased efficiency in the medicines domain would be to put in place regional negotiation mechanisms that could further reduce the price of medicines such as antiretroviral for HIV/AIDS; this would need not only a dialogue within countries but also between countries.

Another key set of issues discussed under this were the inefficiencies related to suboptimal performance of health workers as a result of low motivation and morale among them. The modalities and levels of staff pay for civil servants, as is the case in most contexts in Africa, are largely out of MoH control. This often makes it difficult to implement health financing strategies that could effectively enhance health worker performance through better salaries and other possible financial and non-financial bonuses and/or adjustments. For the ministries of finance (and other relevant ministries overseeing civil service employment and pay) the problem of health worker pay is a question of general public sector salaries and they most often do not wish to see increases in health worker salaries since it can create unbalances (for example very different pay levels say between nurses and teachers) and lead the other civil servant categories to demand similar increases – something that the MoF would tend to see as financially unsustainable in most contexts. However, panel discussion participants from both the MoH and MoF sides seemed to be keen on further developing the different types of result based payment mechanisms that have been now scaled up in some countries to national policies.

It was also emphasized during the meeting that the experiences in result/performance based financing have not been limited to staff incentives and that this approach can also bring system-wide efficiencies depending on the way it is implemented. It was noted that currently the level of evidence on result based financing, while having increased substantially in recent years, was still not adequate and more studies needed to be undertaken.

### Making evidence based health financing decisions

Health financing reforms, actions and interventions cannot happen in a purposeful way without robust, context specific evidence. This was one of the most recurrent crosscutting themes in the discussions between the ministries of health and ministries of finance. The discussions revealed that ministries of health cannot expect to receive more funding for health if they do not demonstrate to ministries of finance that the resources are utilized well and that they produce measurable results. Another key challenge to increasing national health budgets and protecting them during the global economic crisis is the prevailing perception of health as a non-productive sector which does not contribute much to growth and development. This is in part due to the failure of health advocates to demonstrate the positive outcomes of increased health expenditures to MoF. The current unsatisfactory state of health financing was mainly attributed to lack of clear vision and evidence based plans and strategies. Evidence-based planning and budgeting, that includes a clear priority setting aspect was seen as one of the main paths for MoH to convey its resource needs and the objectives it aims to attain with these resources. The output of this process should be a national health strategic plan (NHSP) that is fully costed and that is integrated into the overall development strategy of the country. Linking the NHSP with other development strategies and policies is especially important since it allows framing the dialogue between MoH and MoF (and most often the ministry of planning) around the positive contributions of the health sector to the overall development objectives.

Countries should also build on the national health planning framework and put in place a comprehensive health financing strategy that is guided by a clear vision on how health financing can put countries on the path to universal coverage. These health financing strategies should be outcomes of an internal dialogue between MoH and MoF but also with a much wider group of stakeholders. The national health financing strategy should highlight the roles and responsibilities of all stakeholders in a national, strategic and evidence based approach to health financing for universal coverage. Without a clear health financing strategy, the case for increased spending in health will be weak, since even if the MoF is persuaded by the evidence based case for more investment in health, there will be need for a clear “business plan” that demonstrates how the increased investments will be channeled/allocated and used.

Building evidence was seen as one of the weak points of ministries of health and there was a general call to MoH to produce data and evidence that would be focused on issues of importance to MoF. In other words, there is a need for MoH to speak the language of MoF and thus engage into serious negotiations on financing, priorities and on the outcomes and outputs. This is one of the prerequisites for MoH to get a listening ear from MoF and its engagement in the health financing reforms. Among the important issues is the need for evidence on cost drivers and efficiency improvements in the system. This can reassure the MoF on the steps that MoH can take to address efficiency concerns. At the end of the day, this increased, evidence based, dialogue between MoH and MoF can on the one hand assure the MoF that the money is well used and on the other hand for the MoH to have a solid case to get financial commitments from the MoF. Bringing evidence to the negotiation table can also be a way for MoH to counteract the often prevailing perception of health as a *non-productive* sector which does not contribute much to growth and development. This should make the MoH more attractive for getting more resources from the MoF.

### Key lesson learned

There is surely a long list of things regarding health financing that need to be addressed in most of the African countries on their way towards universal coverage. Many countries still find themselves with several bottlenecks that need a coordinated and strategic approach in order to reform their health financing system. Investing in developing equitable and efficient health financing systems is not only important for putting countries on the path to universal coverage, it is also one of the keys for reaching the MDG targets and other health targets beyond 2015.

The barrier separating MoH from MoF is certainly not the only possible blockage point for effective health financing reforms, but it is one of the most important ones. In order to overcome the possible blockages, a consistent and result driven dialogue between these two ministries is of high importance. The need for close collaboration and policy articulation has been made even more urgent in the aftermath of the 2008 global financial crisis, which has resulted in cost containment and cutting strategies in all government sectors often coupled with reduced aid inflows from development partners.

However, even if there are still challenges regarding this inter-ministerial dialogue, the panel discussions revealed that there are important points of convergences in the discourse and positions of these two entities.^b^ For example, both ministries were keen on focusing on the efficiency aspects of health systems. As the macroeconomic context is still uncertain, the MoH will need to find ways to get more health for the available money. For the MoH, the efficiency question translates to strategic choices such as better-allocation of resources towards prevention and primary care. Another efficiency related area deals with health worker motivation. The World Health Report 2006 “Working together for health” highlighted, among other things, the importance of adequate remuneration and other financial and non-financial incentives [20]. The result based financing mechanisms that have been implemented in several African countries, including pilot projects but also national level programmes, although often considered as system-wide approaches [21], are mainly focused on incentivizing health workers to increase efficiency. Both the MoH and MoF agreed that these approaches can be important drivers for efficiency in the future although more evidence is needed to understand their full potential.

More general for the MoF, efficiency from all its aspects is a question of good use of public resources and accountability. The more the MoF is convinced about the good use of resources in the health sector the more it might be willing to put money in health. A fairly clear consensus coming out of the panel discussions was that the question of efficiency should indeed be a central issue in the dialogues between the two ministries and that there was certainly a lot of common ground on which to build a strategic and ongoing dialogue regarding the best use of resources. The World Health Report 2010 listed ten common areas of health system inefficiency, among others [11]. So it is clear that the demonstration around efficiency needs to take into account several aspects and it will depend to large extent on what are the most important areas of inefficiency in a given country. What is clear is that the discussions between the MoH and MoF on efficiency should build on available evidence on technical efficiency (mainly relating to efficiency at the level of health facility) but go well beyond that with a focus on allocate efficiency – on how different models of investment in health rank between each other but also how health sector investment in general ranks with investment in other sectors.

The panel discussion showed also that some important points of divergence between the two ministries also exist. For example, the question of allocating a larger share of the government budget to health is something where common ground is more difficult to find. This is indeed not a surprise and does of course reflect the mission and objectives of each institution. However, an important lesson learned from the discussions was that some of the current blockage points holding back budget allocations for health can be solved with a more evidence based approach based on a clear national health financing strategy from the MoH side. This should then translate more easily in a language where the MoH’s needs and objectives are effectively conveyed to the MoF. From the health sector point of view one of the key take home messages is thus the need to build capacity in MoH (but also other relevant health sector actors) on generating evidence that is on one hand needed for the strategic planning in health financing but also on the other hand useful regarding the dialogue with the MoF.

## Summary of concluding remarks and next steps to strengthen dialogue between MoH and MoF

Improving health in Africa is a driver for long-term economic growth and development. This linkage of health and wealth is one reason why the ministries of health and finance will need to find common ground on how to create policy coherence and how to articulate their respective objectives. Another reason for the collaboration between these two institutions is the fact that besides agreeing on a vision and objectives for health financing for universal coverage, the actual implementation of reforms and actions to achieve this will need constant dialogue, coordination and a joint leadership.

Universal coverage has been very high on the international agenda recently. How to get there through a strategic approach including an inter-country dialogue has also been discussed in many recent fora. For example, the International Forum on “Sustaining Universal Health Coverage: Sharing Experiences and Supporting Progress”, organized by the government of Mexico in April 2012. This forum gathered ministers and other high-level participants, including from many African countries, resulted in a political Declaration on Universal Health Coverage which noted the need to “work together in our own countries on the development and use of transparent financial mechanisms, accountability and reporting, and monitoring and measuring of health system performance and outcomes. Such actions support the progressive realization of universal health coverage in an efficient, sustainable, and publicly accountable manner”.

The analysis of the panel discussion indicates that there is still room for further improvements in building mutual understanding on some of the key health financing issues and creating a collaborative environment between the MoH and the MoF. The three panel discussions organized to date have already contributed to improving the collaboration between the two ministries and to ensure that optimal funding to the health sector produces desirable results and overall national development.

In order to build on the results of the previous meetings a ministerial conference was organized from 4^th^ to 5^th^ of July 2012 by the Harmonization for Health in Africa (HHA) network in collaboration with the African Union (AU) and the United Nations Economic Commission for Africa (UNECA). This meeting, hosted by the African Development Bank and held in Tunis, Tunisia, provided a platform for enhancing evidence based policy dialogues between MoH and MoF. The meeting provided a unique opportunity for forging consensus regarding the challenges the health sector is facing and for bringing forward successful experiences, from Africa and beyond, in order to find solutions to move African countries towards better health outcomes. The main outputs of this meeting were the Tunis declaration “Value for Money, Sustainability and Accountability in the Health Sector” and the guiding document adoption of “A Country Framework for Enhanced Engagement and Action between Ministries of Finance and Health and their Partners”. Together they provide a roadmap for implementing the outcomes of the dialogue engaged in Tunis, Kampala, Addis Ababa and Yamoussoukro at the country level, based on individual country needs and context. Lastly, in order to coordinate and optimize the mobilization of the international agencies the HHA action plan to support the implementation of the Tunis Declaration was approved by HHA Regional Directors during their annual meeting held in Nairobi 4-5 October 2012.

It is envisaged that the process that has been going on through the past panel discussions and strengthened further during the Tunis meeting would be consolidated at an annual meeting at the level of Ministers of Finance and Health to discuss new high impact investments, review progress on existing programs and propose any needed revisions. The process itself would be reviewed at the same time to ensure that it is relevant and efficient. To ensure transparency and accountability, a summary of the meeting would be provided on relevant government websites.

The international conferences, the panel discussions and all the work around the question of enhancing policy dialogues between MoH and MoF (within a comprehensive stakeholder dialogue) are the first steps in a long process. The next step will consist of feeding the knowledge and the ideas created through this work in the country level processes. Here context is obvious key – every country will need to model the way it wishes to enhance the dialogue process in a way that best fits its politico-administrative environment. However, some basic points on what this country level process would need in order to succeed can be generalized.

More frequent meetings of officials at different levels of the ministries should be held regularly to improve working relations and understanding between the two ministries. This would create mutual understanding on where the two ministries stand on some of the key issues. Once this mutual understanding has been reinforced, the next step is to enhance day-to-day problem solving together.

Finally, the dialogues need to be based on a clear health financing strategy. This strategy can firstly be used for forging some level of common vision; here the concept of universal health coverage will be of great importance in order to focus the dialogues on context specific vision on how to move towards it. Secondly, once the context specific vision has been established, the health financing strategy should be used as a guide to the inter-ministerial dialogues in order to fix the levels of funding and the use of these funds so that the best possible compromise is reached. Once this compromise is reached, the implementation of the strategy will be much easier since it will have a more or less explicit backing of the two ministries – and hopefully of a larger group of stakeholders.

## Statement of ethics

Since the article is based on proceedings meetings where participants took part voluntarily, and did not entail interviewing of human subjects, we assume this article did not require any ethical clearance.

## Endnotes

^a^ The term "financial catastrophe" is often used instead of "severe financial hardship". It is defined technically as spending a disproportionate share of household income (more than 40%) annually on direct payments for health services – WHR 2010.

^b^ We do of course acknowledge that the individuals participating in the panel discussions did not necessarily represent any “official” views of their respective organizations - but for analysis sake and in line with the discourse analysis approach, we have kept the institutional discourse perspective on all the interventions made during the panels.

## Abbreviations

AIDS: Acquired Immuno Deficiency Syndrome; AFR: WHO Africa Regional office; AU: African Union; FDI: Foreign Direct Investment; EMRO: East Mediterranean Regional office; GFATM: Global fund against AIDS TB and Malaria; GGEH: Government general expenditure on health; HHA: Harmonization for Heath in Africa; MGDs: Millennium Development Goals; MoF: Ministry of Finance; MoH: Ministry of Health; NHA: National Health Accounts; NHP: National Health Plan; SWAp: Sector-wide approaches; UNECA: United Nations Economic Commission for Africa; UNICEF: United Nations Children’s’ Fund; UNFPA: United National Population Fund; ODA: Official Development Assistance; OOP: Out of Pocket; PHC: Primary Health Care; SWAp: Sector Wide Approach; THE: Total health expenditure; US$: United States Dollar.

## Competing interests

Authors declare no competing interests.

## Authors’ contributions

LM participated as an observer in all panel discussions, supported synthesis of proceedings and led the drafting of the manuscript. JNO participated as an observer in two of the panel discussions as an observer, supported synthesis of conference proceedings and drafting of the manuscript. RE supported synthesis of conference proceedings and drafting of the manuscript. JK participated as an observer in one of the panel discussion, supported synthesis of conference proceedings and drafting of the manuscript. All authors read and approved the final manuscript.

## Pre-publication history

The pre-publication history for this paper can be accessed here:

http://www.biomedcentral.com/1472-698X/12/30/prepub
